# Anecdote from H. Crane, D. D. S.

**Published:** 1847-10

**Authors:** H. Crane


A lady visited me a short time since, and requested me to replace some four or five plugs, that had rather unceremoniously left as many of her molare teeth. She stated that they were inserted by an excellent dentist, but that she had been so careless as to eat molasses candy, and that it had stuck to the plugs and pulled them out.

Charitable soul! She is certainly worthy of a better planet. I relate the above anecdote of this good old lady, merely to say, that a plug properly inserted cannot be removed from the tooth with anything besides metalic tooth picks, unless the tooth breaks.

H. CRANE.

